# Is the health status of female victims poorer than males in the post-disaster reconstruction in China: a comparative study of data on male victims in the first survey and double tracking survey data

**DOI:** 10.1186/1472-6874-14-18

**Published:** 2014-01-27

**Authors:** Ying Liang, Runxia Cao

**Affiliations:** 1 Department of Social Work and Social Policy, School of Social and Behavioral Sciences, Nanjing University, Nanjing 210023, People's Republic of China; 2 School of Statistics and Mathematics, Zhejiang Gongshang University, Zhejiang 310018, People's Republic of China

**Keywords:** SF-12, HRQOL, Sichuan earthquake, Post-disaster reconstruction, Victims, Female, Comparative study, SEM, Double tracking survey data

## Abstract

**Background:**

The health of females is more at risk during disasters. Studies that focus on the comparison of males and time span are few. This article focuses on the health-related quality of life (HRQOL) of female victims in the post-disaster reconstruction in China. We aim to reduce gender health inequalities by comparing and analyzing gender differences in HRQOL. Moreover, we analyze the trends in HRQOL of female victims by using tracking data, and then provide reasonable suggestions to enhance the HRQOL.

**Methods:**

This article explores the HRQOL of women victims in the post-disaster reconstruction from two perspectives: a comparison between males and a time span of six-month intervals. We conducted the first survey, and the double tracking survey in 2013. This study uses data from half a year later sample surveys collected from five counties (Wenchuan, Qingchuan, Mianzhu, Lushan, and Dujiangyan) in Sichuan in 2013 (N = 2000).

**Results:**

(1) By calculating the Cronbach’s alpha coefficients of the SF-12 scale, we found that that reliability of the scale and the internal consistency are good. (2) Using SF-12 instead of SF-36 to measure the HRQOL of survivors is feasible. (3) The ANOVA and non-parametric testing methods show that significant differences exist between the eight dimensions of HRQOL in different genders after the earthquake. (4) After six months, the HRQOL of female victims in the post-disaster reconstruction has also undergone a significant change. (5) Compared with male victims, we should give more attention to female victims’ HRQOL issues in the post-disaster reconstruction in Sichuan. (6) The performances of victims in the post-disaster reconstruction in PCS and MCS affect each other.

**Conclusion:**

We found that in terms of gender, the male and female victims’ HRQOL after the disaster largely varied: (1) In general, significant difference exists between male and female victims in terms of HRQOL. The HRQOL of female victims is poorer than that of male victims. (2) The PCS and MCS of victims affect each other. However, for female victims, the degree of influence of MCS on PCS is larger than that in males. (3) The MCS of female victims is more vulnerable than that of male victims. In terms of time span, the following information was obtained: (1) after six months of rest, victims’ HRQOL significantly improved. (2) At this stage, relative to the MCS, the PCS of females should be given more attention.

## Background

In the public health of China, women, as a vulnerable group, suffer more severe damage because of personal, social, and health service factors. The vulnerability of their health is made more apparent when emergency situations occur, such as natural disasters. ***Firstly,*** they are at a disadvantage in the face of disasters because of the inequalities in exposure and sensitivity to risk, as well as the inequalities in their accessibility to resources, capabilities, and opportunities [1]. ***Secondly,*** their health is more vulnerable than that of males. Sociologists should always discuss health gender inequality and treat this as an integral part of the design, implementation, tracking, and policy evaluation in the political, economic, and social fields. This consideration would provide equal benefit to females and males [2]. Moreover, a more accurate and complete knowledge can be obtained for fair disaster preparedness, response, recovery, and reconstruction strategy [3].

**Figure 1:**
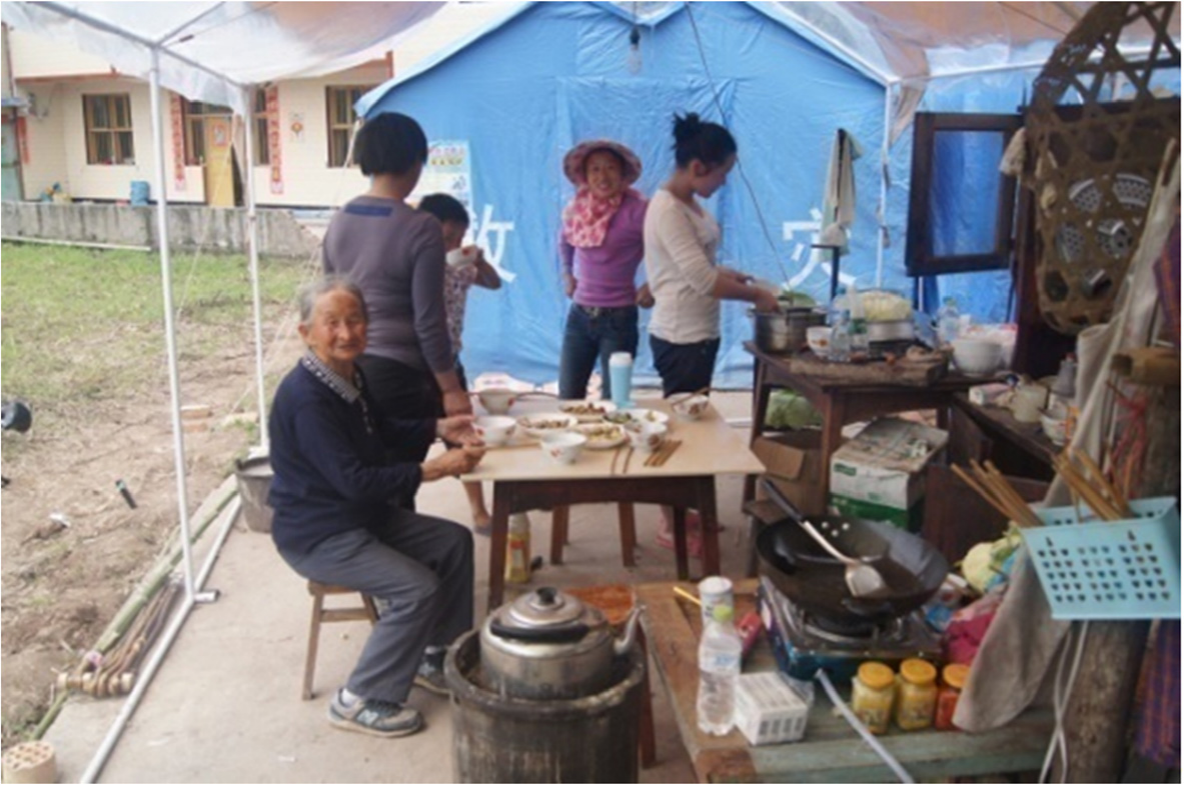
Rural community in Ya’an community.

Therefore, research on female health vis-à-vis gender comparison and tracking is an important field of study. In China, earthquakes pose great risk. Devastating earthquakes in Sichuan, a province in the middle of China, have drawn the attention of the whole country, and even the entire world (Figure 1).^a^ Post-disaster reconstruction is one of the prior tasks of the government. Numerous non-governmental organizations also participate in reconstruction projects. Many health care intervention programs, including emergency relief measures, have been implemented shortly after the earthquake, although the effect of their implementation requires validation. As such, this paper explores the comparison of quality of life (QOL) between males and females after the disaster and analyzes the trends in females’ QOL.

Much concern has been raised with regard to the health of victims. Although many studies on the health of victims are available, most of them have focused on mental illness or post-traumatic stress disorder (PTSD) of victims. Approximately 47.3% of victims are suspected to have developed PTSD in gravely damaged areas, whereas 10.4% [4] in moderately damaged areas. Females are more prone to develop mental illnesses [5,6], such as PTSD, tension, and depression based on surveys conducted several months or one year after the earthquake [7,8]. The risk of illness is high for pregnant females in disaster areas [9]. Moreover, female victims are more likely to develop PTSD compared with male victims [10]. Females exposed to earthquakes have higher risks of PTSD and postpartum depression compared with females who have not experienced earthquakes [11]. In Japan, the long-term suicide mortality rates of females increased after the earthquake, and the suicide mortality rates decreased in males [12]. Based on these data, the psychological condition of female victims is alarming. Adverse mental illnesses, including PTSD and depression, significantly lower the health of victims [13]. However, as time progresses, females gradually forget traumatic memories and begin to look forward to a new life. Moreover, continuous social support results in a more optimistic environment for female victims. Thus, we propose the first hypothesis:

***Hypothesis 1****: The health-related quality of life (HRQOL) of female victims improves after six months of rest.*

HRQOL has received considerable attention in the research community. It is the subset of QOL. QOL has various definitions [14]. It is a multidimensional concept that includes objective and subjective factors, all domains of life, and individual values [15]. Over the past few decades, QOL has attracted research attention. Nevertheless, many studies on the subject need to be enriched and explored [16]. HRQOL is a major branch of QOL, including physical, psychological, emotional, and social functioning aspects. HRQOL focuses on the effect of health status on the QOL of people. The HRQOL data are a vital indicator and reference tool. They establish the communication between patients and clinicians, as well as provide public health policymakers with a meaningful reference [17-20], thereby improving the efficiency of resource allocation [21]. The conventional large-scale measurement of HRQOL is committed to enhancing the efficiency and effectiveness of the healthcare system [17].

HRQOL has also been a pertinent topic when studying the health status of victims [22,23]. Studies on gender differences in the HRQOL of different groups are numerous. A study on the residents of a United States community found [24] that men have significantly better HRQOL than women. For children and adolescents, the HRQOL of boys and girls are roughly the same at a young age. The HRQOL of girls decrease more rapidly than boys with increasing age [25,26]. Studies focusing on the HRQOL of patients with coronary artery disease [27], adolescents with cystic fibrosis [28], and patients with HIV/AIDS are also plenty [29]. The results of these studies suggest that females have worse HRQOL than males.

Unfortunately, studies on the HRQOL of post-disaster victims are limited. Thus far, no study on the difference of HRQOL of earthquake disasters is available. Researchers have considered the research perspective related to HRQOL risk factors. Social support, age, education, and gender are influencing factors of HRQOL [30]. For earthquake victims, old age, being female, economic loss and mental disorders are all risk factors of QOL [31]. In fact, the HRQOL of victims is lower than normal after the earthquake [32]. However, studies that take into account the female gender are few. This lack of data has become our motivation for the present study. Studies have already focused on child and adolescent victims, and the results indicated that the HRQOL of children decreased with the passage of time. The pediatric QOL inventory of girls is lower than that of boys [33]. Therefore, females also need the same attention as children whose HRQOL have been ignored in previous studies. Thus, in the present study, we intend to explore the HRQOL of females in detail.

The vulnerability of female health needs to be investigated more extensively. A large number of female victims, whose number far exceeds that of male victims and whose reproductive health have significantly deteriorated than before the earthquake [34], were taken to the hospital [35]. Moreover, the status of women’s health is in a dire state. Therefore, we propose the second hypothesis.

***Hypothesis 2****: In the post-disaster reconstruction in Sichuan, the HRQOL of both male and female are low, and the HRQOL of females is worse than that of males.*

How about the physical and mental health of the victims in the post-disaster reconstruction? Do males and females exhibit difference in these two areas? As a vulnerable group, females are significantly different from males in terms of physical function and mental endurance. Moreover, the environment of disasters would have changed a lot with the passage of time. Will this change have an effect on the QOL of females? Thus, we propose the third hypotheses in this study.

***Hypothesis 3****: The mental component summary (MCS) of female victims is more vulnerable than that of male victims. Moreover, currently the focus on the HRQOL of females should be changed.*

Monitoring the HRQOL of victims has become increasingly important because of frequently occurring earthquakes. However, studies on the HRQOL of female victims do not exist. As such, this study uses the 12-item short form health survey (SF-12) to measure the HRQOL of victims. The objectives of this study are as follows: (1) provide basic information on the HRQOL of victims in post-disaster reconstruction, (2) analyze the gender difference in the post-disaster HRQOL of victims, (3) compare the HRQOL data, which were classified into two groups, and analyze the trends, (4) provide suggestions for government and social organizations in improving the HRQOL of female victims.

## Methods

### Participants and procedures

The data were obtained from two sample surveys on five counties in Sichuan in May and June, 2013, which was classified into two groups. The multi-stage sampling method was mainly used. First, we selected five cities (Aba, Guangyuan, Deyang, Ya’an, and Chengdu) from 39 hard-hit areas via the simple random sampling method. We selected one county in each city (Wenchuan, Qingchuan, Mianzhu, Lushan, and Dujiangyan, respectively) via the simple random sampling method. Finally, we randomly selected 400 victims in one county via the quota sampling method. Four of the selected counties, namely, Wenchuan, Qingchuan, Mianzhu and Dujiangyan, were hard-hit areas during the Wenchuan earthquake in 2008. As a result, tens of thousands of people were found dead, missing, or injured. Then, coincidentally, an earthquake with 7.0 magnitude hit Ya'an, one county of the frequently stricken areas in Sichuan in April 20, 2013, resulting in 196 deaths, 21 missing, and 11,470 injured.

The questionnaires were mostly distributed in the placement cell built for victims after the earthquake. Each participant independently completed the questionnaire based on instructions of the investigators. A total of 2000 questionnaires were distributed, and 1672 were received. The response rate was 83.6%. In the questionnaire, we asked respondents to provide their contact information, including fixed telephone, mobile phone number, and address to track the survey respondents.

The second survey was conducted in October and November 2013, and the main method was through telephone survey and household survey. The telephone survey method has many advantages, including low cost, wide coverage, and fast response rate [36]. In other countries, telephone surveys are widely used to perform follow-up investigation of public health cases [37-40]. In China, the use of computer-assisted telephone interviewing (CATI) is very popular in follow-up surveys because of its low cost [41]. Filling out the SF-12 scale through phone investigation is permitted [42].We first performed a return visit based on the information gathered in the first survey. We performed the household survey if we failed to contact the respondents via telephone. The second tracking of the respondents resulted in 1526 completed surveys, and the effective tracking rate was 91.3%. About 53% of respondents completed the survey via telephone and the other 47% via household survey. The causes of sample loss included erroneous telephone numbers and addresses, death, unavailability because of work, change in location, and unwillingness to accept the re-investigation.

### Instruments

The questionnaire focused on the post-disaster HRQOL of victims in Sichuan. In this study, the measurements included two aspects, namely, basic information and the SF-12 scale.

(1) Basic information: gender, age, education, marital status, and monthly income. This study only chose gender variable as the object of study.

(2) The SF-12 scale: The SF-12 scale is a subset of the 36-item short form health survey (SF-36) scale. The original purpose of developing the SF-12 scale is to create a brief generic health status measure that can reproduce the scores of the SF-36 scale [43]. Both have eight dimensions, namely, physical function (PF), role physical (RP), bodily pain (BP), general health (GH), vitality (VT), social function (SF), role emotional (RE) and mental health (MH). The eight domains can be divided into two main domains: PCS and MCS. PCS includes PF, RP, BP, and GH, whereas MCS includes VT, SF, RE, and MH.

Studies have shown that the SF-12 scale is suitable for the evaluation of QOL of the Chinese population, including children [44,45] and migrants [46]. This suitability was also confirmed in the evaluation of QOL of earthquake-stricken residents. The method has good reliability and validity [47]. In addition, the SF-12 scale is more sensitive in evaluating the health burden of recent health problems and social inequalities [48]. The method also has good reliability and validity in follow-up investigations [49].

The scores measured by SF-12 and SF-36 are highly related and similar [50], such as patients with and without obesity [51] and those that need dialysis [52]. SF-12 and SF-36 have a similar prognostic association with death and hospitalization risk. However, SF-12 has smaller loss (10%) in the ability to distinguish between different disease groups than SF-36. Nonetheless, SF-12 remains an effective alternative to SF-36 [53]. SF-12 has been tested by different researchers in different patient groups, such as patients with coronary heart disease [53], stroke [54], and chronic pancreatitis [55].

In addition, we found that the enthusiasm of respondents to fill out the questionnaires decreased during the tracking investigation. Thus, a questionnaire with fewer items can ease their weariness. Based on the literature review, SF-12 has good validity and reliability and is an effective substitute. Therefore, we propose the following idea: Using SF-12 in the large-scale tracking investigation is more practical and effective, although the scores can maintain the measurement effect of SF-36.

### Analysis methods

(1) Reliability analysis

To test the reliability and effectiveness of our questionnaires, we conducted reliability analysis, from which we can determine if the instruments used are appropriate. Many methods to test the reliability are available. We used the Cronbach’s α(alpha) coefficient, which is a commonly used method. A Cronbach’s α coefficient larger than 0.5 indicates a reliable questionnaire.

(2) Analysis of variance

ANOVA is a method used to test if the mean differences between two or more objects are statistically significant. To make ANOVA more effective, the variance between different objects is usually assumed to be the same. Differences in the HRQOL between male and female victims will be discussed. Thus, the homogeneity of variance of all subjects will be tested first, and ANOVA will be employed thereafter.

(3) Non-parametric test

The nature of objects in which the samples belong to should be considered when ANOVA is used. Thus, particular limitations need to be considered for the distribution shape of objects. ANOVA is not suitable when the variance between male and female objects in one dimension is unequal. The overall parameter is not included in non-parametric tests. Thus, the testing conditions of this method are relatively loose and adaptable. The non-parametric test is also flexible and widely used. The non-parametric test used in the following analysis is the Mann–Whitney U test. The test is deduced by ranking the average of two group samples. The original hypothesis of the Mann–Whitney U test is that the overall distribution of two group samples has no significant difference.

(4) Structural equation modeling

Structural equation modeling (SEM) has been widely used in various fields of social sciences. SEM not only estimates the relationship among observed variables, representative latent variables, and factors in a group but also analyzes the relationships between latent variables. SEM is a multivariate statistical technique for testing the hypotheses in terms of the influences of sets of variables on other variables. These hypotheses can involve correlational and regression-like relations among observed and latent variables [19]. SEM mainly includes two parts, namely, confirmatory factor analysis and structural equation (SE). SEM can assess the relationship between latent variables. In this study, we need to construct SEs to analyze the eight dimensions of HRQOL in the SF-12 scales.

### Ethical statement

Before respondents filled in the questionnaire in this study, they had been told that their data would be used for academic research, and they ensured that their information filled in the questionnaire was in accordance with the actual situation. The interviewers are well trained to improve the quality of their data collection skills and meet the requirements of the medical ethics.

The survey conducted with oral informed consent and the approval of the ethics committee of the School of Social and Behavioral Sciences Nanjing University, in compliance with the principles of the Declaration of Helsinki. Interviewers informed each respondent of their right to refuse to participate, and of their right to refuse to answer any question, both initially and during the course of the research. After making sure that the respondents were clear about their rights and the possible consequences of this study, the interview would start. And authors would take the interpretation and responsibility for results involving human subjects in this study.

## Results

### Reliability analysis of SF-12 scale

In this paper, we used the first survey to analyze data reliability. Table 1 shows that each Cronbach’s α coefficient in the five scales are almost larger than 0.800, which indicates that the scales have good internal consistency and reliability. The overall Cronbach’s α coefficient is 0.828, which indicates that the general reliability of the questionnaire is also good and that the questions in this questionnaire show strong internal consistency. The second column of Table 1 shows the total mean of the remaining variables after removing one item. For example, the total mean of the seven remaining variables after removing VT is 295.16. It is the smallest number in the first column, which indicates the score of VT and has a greater effect on the total mean. The third column shows the scale variance of the remaining variables after removing one item. The fourth column shows the total correlation coefficient between one item and the other items. The correlation coefficient between MH and the other seven items is 0.684.

**Table 1:** Reliability analysis of the SF-12 scale

	**Total mean if item is removed**	**Scale variance if item is removed**	**Total correlation if item is removed**	**Cronbach’s α coefficient if item is removed**
PF: physical function	300.67	30186.165	0.607	0.802
RP: role physical	301.61	28727.631	0.563	0.807
BP: bodily pain	299.19	29610.768	0.526	0.812
GH: general health	305.63	29575.140	0.526	0.812
VT: vitality	295.16	29598.358	0.525	0.812
SF: social function	299.58	30224.805	0.500	0.815
RE: role emotional	299.28	28994.808	0.541	0.810
MH: mental health	302.80	30257.810	0.684	0.796

### Comparison of measurement effect between SF-12 and SF-36

The SF-36 scale has numerous items. Thus, the respondents were impatient to finish the scale and hoped to fill out a brief questionnaire. In this section, we explore the relationship between the eight domains of SF-12 and SF-36 to determine whether the similarity of measurement scores between the two scales is sufficiently high.

**Figure 2:**
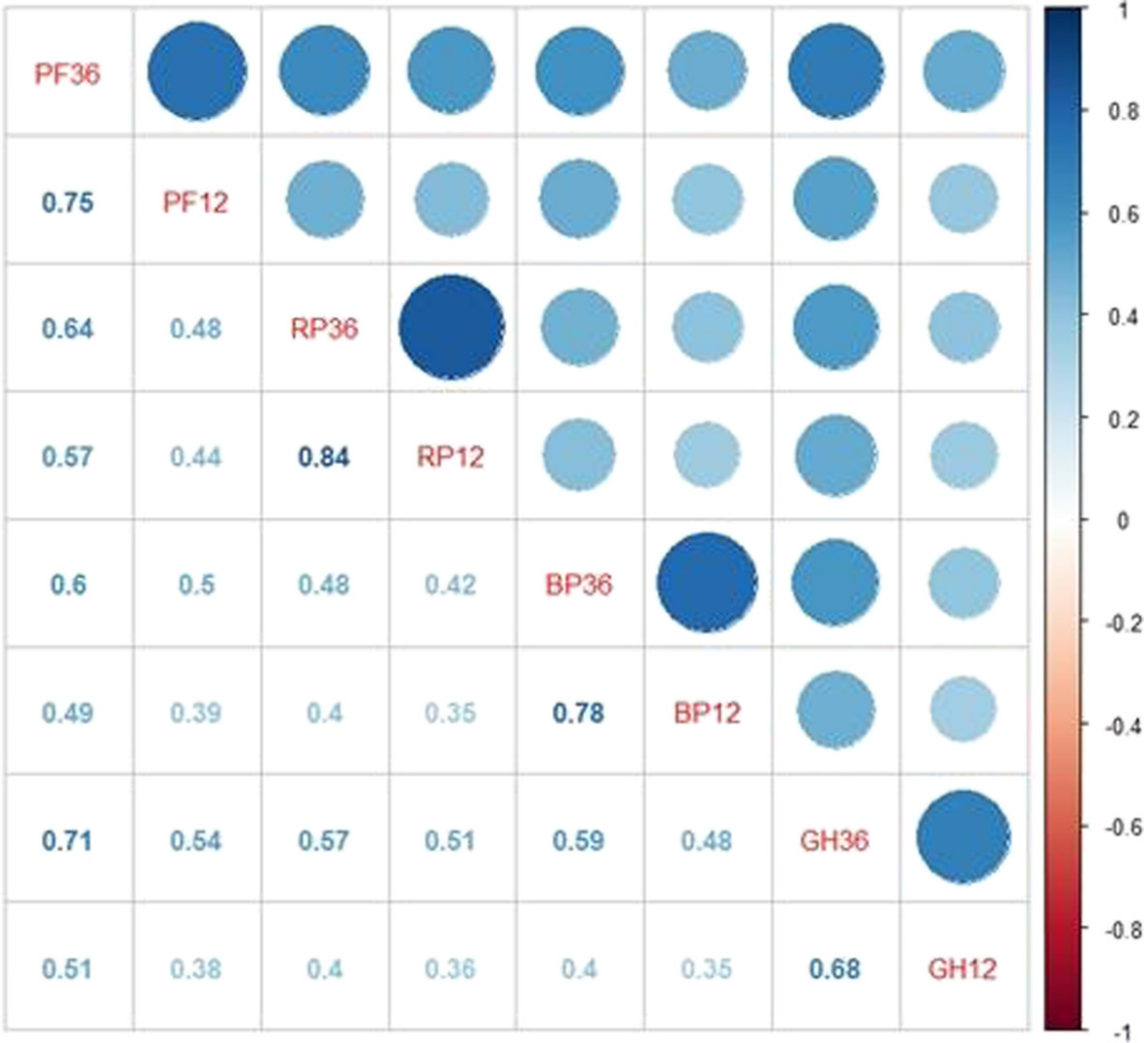
Correlations between four domains of PCS of two scales.

Figure 2 shows the relationship between the four domains of PCS (PF36 indicates the PF variable of SF-36, whereas PF12 indicates the PF variable of SF-12; thus, we can interpret other items in a similar way). The darker and larger the circle is, the greater the correlation coefficient is. The lower left corner represents the value of the corresponding correlation coefficient. The first line indicates the correlations between PF36 and seven other variables. As shown, the correlation coefficients of the four groups of variables (PF36 and PF12, RP36 and RP12, BP36 and BP12, and GH36 and GH12) are relatively high, that is, 0.75, 0.84, 0.78, and 0.68, respectively. Thus, the correlations of PCS are relatively high.

**Figure 3:**
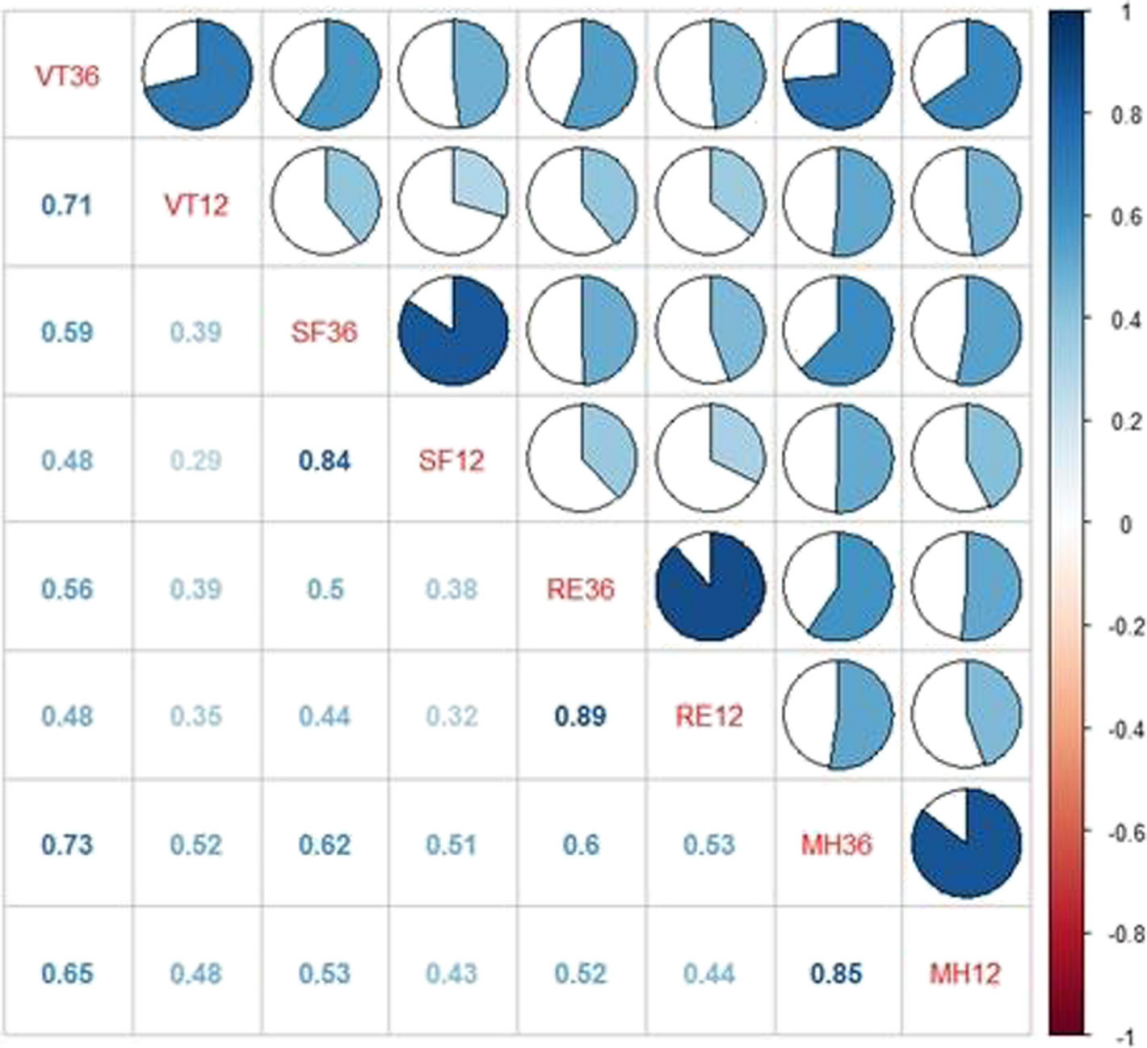
Correlations between four domains of MCS of two scales.

Figure 3 illustrates the relationship between the four domains of MCS. The more shaded the cells are, the greater relevance they indicate. The correlation coefficients of the four groups of variables of MCS (VT36 and VT12, SF36 and SF12, RE36 and RE12, and MH36 and MH12) are relatively high, that is, 0.71, 0.84, 0.89, and 0.85, respectively. Thus, the correlations of MCS are relatively high.

Therefore, according to the comparison of two domains of two scales, we concluded that using SF-12 instead of SF-36 to measure the HRQOL of survivors is feasible. This can make the investigation more simple and efficient.

### Study on the difference of HRQOL among genders and tracking data

ANOVA tests whether the mean differences between two objects are statistically significant. Thus, homogeneity of variance was tested to determine if the variances of different genders and points are homogeneous. We determined if comparative analysis can be conducted via one-way ANOVA.

Table 2 shows the test results for homogeneity of variances. Compared with males’ data, only the probability (P) value of VT is larger than 0.05, which indicates that the variances of VT and SF with different genders are the same and meet the prerequisite of ANOVA. However, the P-value of the remaining seven variables is lower than the significant level, which indicates that variances with different genders are different, and ANOVA is not suitable for these variables. Compared with the tracking data, the probability (P) values of PF, RP, GH, VT, and RE are larger than 0.05, and ANOVA is suitable. Thus, one-way ANOVA is used to test whether genders show significant differences with VT and whether different times show significant differences with PF, RP, GH, VT, and RE. Meanwhile, the multi-independent sample testing method in the non-parametric test was used to test whether other variables show significant differences.

**Table 2:** Test results for homogeneity of variance

	**Compared with males’ data**				**Compared with tracking data**			
	**Levene statistics**	**Df1**	**Df2**	**Sig**	**Levene statistics**	**Df1**	**Df2**	**Sig**
PF: physical function	10.757	1	1670	0.001	3.571	1	1637	0.059
RP: role physical	7.630	1	1670	0.006	0.670	1	1637	0.413
BP: bodily pain	10.994	1	1670	0.001	8.671	1	1637	0.003
GH: general health	35.362	1	1670	0.000	2.411	1	1637	0.121
VT: vitality	.232	1	1670	0.630	0.811	1	1637	0.368
SF: social function	5.923	1	1670	0.015	706.480	1	1637	0.005
RE: role emotional	7.329	1	1670	0.007	534.433	1	1637	0.907
MH: mental health	63.406	1	1670	0.000	9.469	1	1637	0.002

Compared with the males’ data, the deviation sum of squares of VT is 2357300.478 (Table 3). However, if we only consider the influence of a single factor, then the total variation in the field of physiology is 225336.870. The variation caused by sampling error is 2131963.609, and the variances are 225336.870 and 1276.625. After division, the resulting F statistic is 176.510, and the corresponding probability P-value is approximately 0. Therefore, the null hypothesis is rejected if α is 0.05. After analysis, we considered that the different effects of gender on VT are significant. Similarly, for comparison with the tracking data, we conclude that the different effects of time on PF, RP, and RE are significant and those on GH and VT are not significant.

**Table 3:** Test results for ANOVA

			**Sum of squares**	**df**	**Mean square**	**F**	**Sig**
Compared with males’ data	VT	Inter-group	225336.870	1	225336.870	176.510	0.000
		Intra-group	2131963.609	1670	1276.625		
		Total	2357300.478	1671			
Compared with tracking data	PF	Inter-group	5274.302	1	5274.302	6.578	0.010
		Intra-group	1312564.319	1637	801.811		
		Total	1317838.621	1638			
	RP	Inter-group	6546.093	1	6546.093	5.285	0.022
		Intra-group	2027648.538	1637	1238.637		
		Total	2034194.631	1638			
	GH	Inter-group	1748.215	1	1748.215	1.491	0.222
		Intra-group	1919965.482	1637	1172.856		
		Total	1921713.697	1638			
	VT	Inter-group	3640.630	1	3640.630	2.903	0.089
		Intra-group	2052823.067	1637	1254.015		
		Total	2056463.697	1638			
	RE	Inter-group	22386.588	1	22386.588	17.289	0.000
		Intra-group	2119602.429	1637	1294.809		
		Total	2141989.018	1638			

Based on Table 4, whether in gender or different time points, differences on other remaining factors are significant. Combined with the above analysis of the variance table, we can deduce the following: 1. the gender differences in the eight dimensions of HRQOL are significant, so comparing the HRQOL of victims under different genders is rational; 2. in addition to GH and VT, the difference on the other six dimensions of SF12 scale at different time points is significant. As most dimensions exhibit differences, comparing the HRQOL of female victims at different time points is also logical.

**Table 4:** Test results for non-parametric tests

	**Compared with males’ data**						
	**PF**	**RP**	**BP**	**GH**	**SF**	**RE**	**MH**
Mann–Whitney U	208955.5	230253.5	233652	234420	238673.5	231511.5	196786.5
Wilcoxon W	583500.5	604798.5	608197	608965	613218.5	606056.5	571331.5
Z	-14.563	-12.869	-11.997	-12.093	-11.452	-12.694	-15.532
Asymp. Sig.	0	0	0	0	0	0	0
	**Compared with tracking data**						
	**BP**			**SF**		**MH**	
Mann–Whitney U	311452.000			312502		314984.000	
Wilcoxon W	685997.000			687047.000		689529.000	
Z	-2.526			-2.407		-2.090	
Asymp. Sig.	0.012			0.016		0.037	

**Figure 4:**
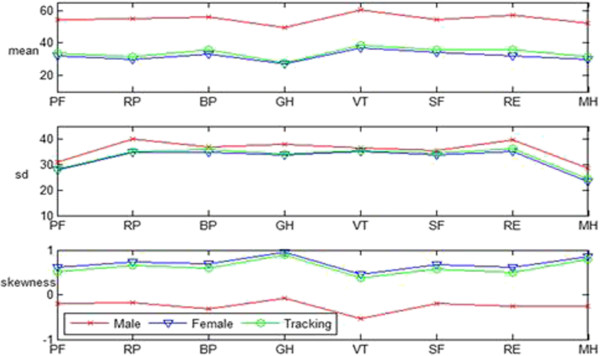
Comparison of the eight dimensions of HRQOL.

Figure 4 compares the basic descriptive statistics information of the samples of males’ data (807 samples), females’ data (865 samples), and female tracking data (774 samples) in the eight dimensions of the SF-12 scale. The mean, standard deviation, and comparison of skewness are given from top to bottom. The mean is often used to present the general level of the statistical objects. This statistics is used to describe the concentration degree of data. Standard deviation is the average distance that each data deviates from the mean and can reflect the discrete degree of a data set. Skewness is the statistics used to describe the distribution pattern symmetry of variable values. If the skewness value is larger than zero, the distribution pattern is skewed to the right. The pattern is skewed to the left when the skewness value is less than zero. Normal distribution has zero skewness. The larger the absolute value of the skewness, the larger the skew degree of the data distribution pattern.

Figure 4 shows that the mean value of males and females in each dimension are small, wherein the mean value for the females is significantly lower than that for the males. These data show that the PCS and MCS of the victims are vulnerable. Furthermore, the HRQOL of the female victims is worse than that of the male victims, and the standard deviation of the female subjects is smaller than that of the male subjects. These findings indicate that the HRQOL of females is relatively concentrated. Figure 4 also shows that the skewness value of the female subjects is larger than zero, indicating that the distribution pattern for this group is skewed to the right. By contrast, the male subjects show the opposite. The skewness value indicates that most females scored lower than their mean. Only few female victims obtained scores higher than their mean. This finding is opposite that of males. The above descriptive analysis indicates that the overall HRQOL of female victims in the post-disaster reconstruction is poor in relation to that of the males.

In terms of the length of time from the earthquake, we found two significant differences when investigating the HRQOL of females as follows: 1. For the mean, eight dimensions of SF12 increased; 2. For the standard deviation, eight dimensions of SF12 increased, but were not very significant; 3. For the skewness, although the skewness of the eight dimensions still skewed to the right, the skewness declined. These findings indicate that the HRQOL of females in the post-disaster reconstruction improved after six months of rest.

In the next section, the different post-disaster HRQOLs of males and females and the different post-disaster HRQOL of female victims at different times will be analyzed using SEM.

### SEM analysis

**Figure 5:**
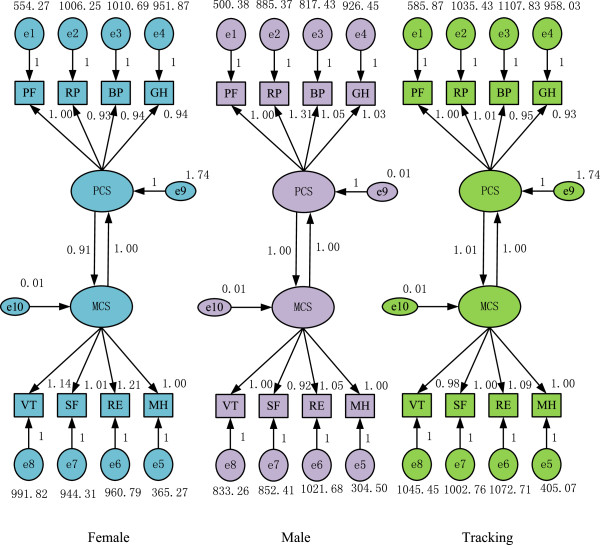
SEM comparison (left: female; middle: male; right: tracking data of female).

The proportion of PCS and MCS in the SF-12 scale will be compared by SEM to determine whether PCS and MCS differ according to gender at different times. Table 5 shows the goodness of fit for SEM on the HRQOL of the female victims in the post-disaster reconstruction. Figure 5 shows the corresponding SEM path coefficient map, which shows the path coefficients corresponding to the survey data of the female victims, the path coefficients corresponding to the survey data of the male victims, and the path coefficients corresponding to the tracking data of the female victims.

**Table 5:** Summary tests on overall goodness of fit for SEM on the HRQOL of victims

**Statistical testing index**	**Standard or critical value for adaption**	**Females’ data**		**Males’ data**		**Tracking data**	
		**Test results**	**Adapted model**	**Test results**	**Adapted model**	**Test results**	**Adapted model**
Absolute index for goodness of fit							
RMR	<0.05	20.946	No	28.627	No	28.775	No
RMSEA	<0.08	0.000	Yes	0.027	Yes	0.012	Yes
GFI	>0.90	0.995	Yes	0.990	Yes	0.993	Yes
AGFI	>0.90	0.991	Yes	0.982	Yes	0.987	Yes
Added index for goodness of fit							
NFI	>0.90	0.975	Yes	0.984	Yes	0.966	Yes
IFI	>0.90	1.005	Yes	0.994	Yes	0.996	Yes
TLI(NNFI)	>0.90	1.007	Yes	0.992	Yes	0.995	Yes
CFI	>0.90	1.000	Yes	0.994	Yes	0.996	Yes
Simple index for goodness of fit							
PGFI	>0.50	0.553	Yes	0.577	Yes	0.579	Yes
PNFI	>0.50	0.697	Yes	0.738	Yes	0.724	Yes
PCFI	>0.50		Yes	0.745	Yes	0.747	Yes
	0.714						

Table 5 shows that most indexes for the goodness of fit reached an acceptable standard, indicating that the model fits the data well. Thus, the external quality and the convergent validity of the model is good. This model is acceptable.

The P-value of the path coefficient of the model is less than 0.001, as indicated by the significance test. Figure 5 shows that for different genders, (a) the factor loadings of the four domains of PCS are not that different from those of the MCS. (b) The correlation coefficients between PCS and MCS were high and exhibited significant relevance, which indicates that strong interactions exist between PCS and MCS. For different times, (a) whether PCS or MCS, the ratio of the gap between the various dimensions exhibited a decreasing trend and (b) the relative degree of interaction between PCS and MCS presented a discrepancy. To facilitate comparison, the path coefficient was changed into the composition ratio, as shown in Table 6. By comparison, we found the different performances of the HRQOL of the female victims on gender and time.

**Table 6:** Composition ratios of PCS and MCS on gender difference

	**PCS**				**MCS**			
	**PF**	**RP**	**BP**	**GH**	**VT**	**SF**	**RE**	**MH**
Female	26.25%	24.41%	24.67%	24.67%	26.15%	23.17%	27.75%	22.94%
Male	23.31%	28.21%	24.48%	24.01%	25.19%	23.17%	26.45%	25.19%
Tracking	25.19%	25.44%	24.69%	24.69%	24.08%	24.57%	26.78%	24.57%

**Figure 6:**
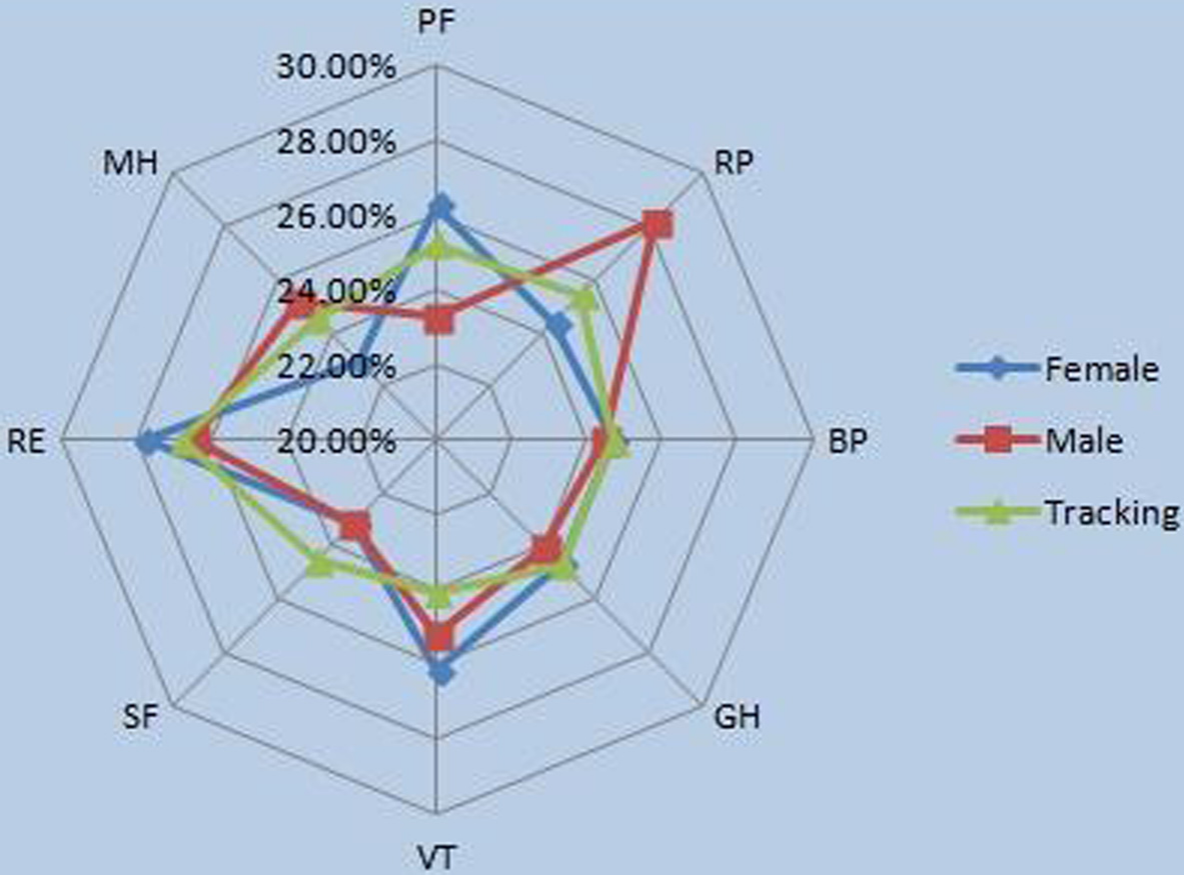
Rader chart of composition of HRQOL.

The proportional distribution of the four dimensions of PCS of female victims is more concentrated than that of the males. As Table 6 and Figure 6 indicate, (1) the proportion difference of the four dimensions of PCS of the female victims is smaller than that of male victims. (2) The PF of the female victims occupies a relatively large proportion (26.25%) in the PCS, and the differences in the remaining variables are small. (3) Significant differences exist in the proportion of the four dimensions of the PCS of the male victims, in which RP has the largest proportion (28.21%). PF has the smallest proportion (23.31%).

The degrees of importance of the four dimensions that affect the PCS of both male and female victims post-disaster vary. The most significant factor of PCS for the female victims is PF, followed by BP and GH, whereas the least significant factor is RP. However, the order for male victims is RP, BP, GH, and PF. The above description indicates that for female victims, PF has the highest significance among the PCS. Post-disaster, daily activities, such as moderate exercise and climbing stairs, are restricted in females. On the contrary, the influence of the PF of male victims is the smallest, but that of RP is the greatest.

The proportional distribution of the four dimensions of PCS for the female victims is different from that of the male victims. Specifically, (1) the most significant MCS for the female victims is RE. Thus, work and daily activities of females are affected by emotional problems. This factor is followed by VT, SF, and MH. For male victims, VT has the highest proportion, followed by VT (51.64%). However, the VT of the males is smaller than that of the females (54%). (2) In the four dimensions of PCS, MH has the largest gender difference. In general, MH relates to the psychological status of a person, e.g., whether he is calm or undergoes emotional depression.

Interaction differences exist between MCS and PCS. Figure 5 shows that for female victims, if the influence of MCS on PCS is set as 1, the influence of PCS on MCS will be 0.91. This finding illustrates that the influence of MCS on PCS is larger than the influence of PCS on MCS. For male victims, both the influence of MCS on PCS and the influence of PCS on MCS is 1, which indicates that they have the same degree of influence.

For different times, the ratio that constitutes the four dimensions of PCS and MCS presents a decreasing trend. Each ratio is closer to its theoretical value (25%). Specifically, the proportion ratio range of the female data that constitute the four dimensions of PCS in the first survey is 1.84% (= 26.25%–24.41%), whereas the tracking data is 0.75%. Meanwhile, the proportion ratio range of the female data that constitute the four dimensions of MCS in the first survey is 4.81%, whereas the tracking data is 2.70%.

For different times, the relative degree of interaction between PCS and MCS presents a discrepancy. In the first survey, female data MCS has a greater influence on PCS than PCS to MCS, whereas that of the tracking data exhibits the opposite trend. This finding may be in line with the actual situation. Shortly after the earthquake, the victims suffered from a greater degree of psychological than physical trauma. In HRQOL in the post-disaster reconstruction, MCS is dominant in influencing the PCS. With the passage of time, the victims have slowly come to terms with the disaster and gave more attention to their health. The condition of PCS, whether good or bad, will greatly affect MCS.

## Discussion

Sichuan enjoys the reputation of being “the land of abundance” in China, but people are suffering from the damage caused by the recent earthquake. Post-disaster reconstruction has to focus not only on the fixed target, such as economic recovery and social order, but also on the soft index, such as the health and QOL of survivors. These elements reflect whether the reconstruction effect can help recover the physical and psychological health of survivors or not. In the face of the earthquake disaster, female groups’ health vulnerability issues are urgent and important. This article focuses on female victims’ HRQOL after an earthquake disaster. To gain insight into females’ health statuses and the changes that ensue after a particularly severe earthquake, we compared the former with the health statuses of males and performed a tracking survey with an interval of six months. Through analysis and modeling, we have obtained the following findings:

(1) By calculating the Cronbach’s alpha coefficients of the SF-12 scale, we found that that reliability of the scale and the internal consistency are good.

(2) Using SF-12 instead of SF-36 to measure the HRQOL of survivors is feasible.

(3) The ANOVA and non-parametric testing methods show that significant differences exist between the eight dimensions of HRQOL in different genders after the earthquake.

(4) After six months, the HRQOL of female victims in the post-disaster reconstruction has also undergone a significant change. Comparing the two sets of female survivor survey data, the ratio of the gap of two aspects and eight dimensions of PCS and MCS that constitute HRQOL present a decreasing trend, which shows that the female victims’ HRQOL significantly improved. Hypothesis 1 is thus supported.

(5) Compared with male victims, we should give more attention to female victims’ HRQOL issues in the post-disaster reconstruction in Sichuan. Whether in terms of overall average effect or data distribution, females’ PCS and MCS are worse than those of males. Therefore, compared with that of males, the HRQOL of female victims should receive more attention. The author believes that although the ratio of female victims’ RP in the PCS is smaller than that of men, it does not indicate better performance in this aspect. However, because of physical problems, female fitness is worse than that of male after the earthquake affected the PF of the female victims. In this respect, males are less affected. Thus, hypothesis 2 is supported.

(6) The performances of victims in the post-disaster reconstruction in PCS and MCS affect each other. For female victims, the degree of influence of MCS on PCS is larger than that in male victims. Relative to male victims, we should give more attention on females’ MCS problems. Females’ MCS are more vulnerable, and the degree of influence of MCS on PCS is larger. Thus, in the post-disaster reconstruction, we should not only solve the physiological damage caused by disaster on females, but also their spiritual problems. At this stage, to improve the female victims’ HRQOL, we can gradually shift focus from MCS to PCS. For female victims, when the earthquake had just occurred, their HRQOL were greatly affected by MCS. In addition, the average female’s physique is relatively poor, so PCS suffered greatly. When the MCS has been resolved, the problem of PCS is gradually highlighted. Therefore, hypothesis 3 is supported.

As evident in the second conclusion, the HRQOL of all victims in the post-disaster reconstruction in Sichuan is poor. Females scored lower than males in both PCS and MCS. On one hand, this finding validates our hypothesis that the victims had poor HRQOL. In fact, a previous study has emphasized the importance of health inequalities between males and females [56]. Numerous reasons, such as social capital, roles, health facilities, and economic and cultural influences, can cause these inequalities and lead to the complexity of many social issues [57-59]. A study on the EQ-5D population in 2008 found that women had poorer HRQOL than men. This conclusion is consistent with ours [60]. This result denotes that more attention and support should be given to the HRQOL of victims, particularly females. The conclusion also confirmed our concerns on the HRQOL of females.

We also found that the MCS issues of females are more significant than those of males. ***First,*** we found that for females, the degree of influence of MCS on PCS is larger than that in males. The reason for this phenomenon is that in terms of the total population characteristics, males and females have different types of mental illnesses. Females have more internal disorders than males, and can easily turn problematic feelings into depression and anxiety [61]. To a certain extent, this difference may explain why the degree of influence of MCS on PCS in females is larger than that in males. Psychological factors have important functions in regulating the normal physiological functions of the human body. The recurring negative emotions can cause long-term or excessive mental tension and give rise to internal problems as vegetative dysfunctions or endocrine dysfunctions. Internal problems may also increase blood pressure, and in severe cases, may even cause some organ or system diseases. Therefore, the physical functions of females cannot be ignored because they are inclined to suffer from internal disorders.

***Second,*** compared with male victims, the MCS of female victims is more vulnerable, which corresponds to a previous study on the HRQOL of earthquake victims in Taiwan. A previous study showed that the PCS of male and female victims did not differ significantly. However, in terms of MCS, females scored significantly lower than xmales [31]. Females had risk factors associated with developing mental illnesses, such as PTSD, tension and depression [5-8]. Their mental capacity in dealing with disasters is weaker than that of males because females have to face dual pressures from living and working, especially the conflict of multiple social roles and gender culture extrusion. Females suffer from natural factors, such as physiology and age, and life factors, such as marriage, child-rearing and family. Therefore, in post-disaster reconstruction, we should not only solve female physiological damage caused by disasters but also female spiritual problems. We should also focus on the MCS of females.

Females, as a large vulnerable group, need more attention and help than males do. Special psychological interventions should be offered during the recovery and reconstruction post-disaster phase to help females who lost their families and homes rebuild their social relationships, adapt to the new environment, and have appropriate understanding of their individualities. In this respect, we can start from the traditional culture and system; integrate community, group, and casework methods; and provide support from the level of communities, families, groups and individuals.

Specifically, ***first,*** at the community level, the following could be mobilized: community-based organizations, community education, community care, and other projects to provide support for females. At the same time, social groups should be actively involved in volunteering, civil society organizations, and community programs to incorporate professional skills and integrate various social resources [62]. ***Second,*** at the family and community level, parent–child relationships, family relationships, female groups, health checks, and mutual self-help groups should be facilitated and enhanced to provide support. Apart from using traditional psychological treatments, such as psychological clinics, group communications, and hotlines, we should also take full advantage of live television, the Internet, and other new media. ***Third,*** at the individual level, the following could be undertaken: financing of poor families, women counseling, elderly care, and providing general support. In the implementation of these policies, special attention should be given to gender-sensitive factors. Support projects should be combined with the characteristics of the women in the community.

Finally, this paper has some limitations. ***First,*** tracking survey in this paper is based on a six-month interval, but the influence of earthquake on victims’ HRQOL may be profound. Future studies could extend the time of tracking. ***Second,*** in China, aside from the earthquake in the central provinces, typhoons in southeastern provinces, as well as floods and drought in the Northwest Territories are all tremendous natural disasters. Some people in these areas are also affected by these disasters. Future research could be extended to other victims’ HRQOL after natural disasters. ***Third,*** the paper focuses on the comparison of female and male HRQOL, as well as the changes exhibited over time. However, the reason for such changes and differences remains unclear.

## Conclusions

In this paper, we present the findings of a comparison of female and male HRQOL after the tremendous earthquake in Sichuan China, as well as the results of a tracking survey after six months. We found that in terms of gender, the male and female victims’ HRQOL after the disaster largely varied: (1) In general, significant difference exists between male and female victims in terms of HRQOL. The HRQOL of female victims is poorer than that of male victims. (2) The PCS and MCS of victims affect each other. However, for female victims, the degree of influence of MCS on PCS is larger than that in males. (3) The MCS of female victims is more vulnerable than that of male victims.

Thus, we should focus on the MCS of female victims in the post-disaster reconstruction in Sichuan. In terms of time, the following information was obtained: (1) after six months of rest, victims’ HRQOL greatly improved. (2) At this stage, relative to the MCS, the PCS of females should be given more attention. Thus, we posit that appropriate social interventions, including psychological and physical support, should be given to female earthquake victims. Meanwhile, to further improve female victims’ HRQOL in the post-disaster reconstruction, we can provide targeted measures after considering the characteristics of females.

### Endnotes

^a^Pictures were taken in Ya'an, one of the survey sample places, during the investigation shoot. The people in the image have consented to publish this picture.

## Abbreviations

HRQOL: Health-Related Quality of Life; PTSD: Post-traumatic stress disorder; SF-12: The 12-item short form health survey scale; PCS: Physical Component Summary; MCS: Mental Component Summary; CFA: Confirmatory Factor Analysis; SEM: Structural Equation Models; PF: Physical Functioning; RP: Role Physical; RE: Role emotional; SF: Social Functioning; BP: Bodily Pain; GH: General Health; VT: Vitality; MH: Mental Health.

## Competing interests

The authors declare that they have no competing interests.

## Authors’ contributions

YL wrote and revised the manuscript, was responsible for the design of the study, and performed the statistical analysis. RC participated in the statistical analysis. Both authors read and approved the final manuscript.

## Pre-publication history

The pre-publication history for this paper can be accessed here:

http://www.biomedcentral.com/1472-6874/14/18/prepub
